# Implementing Social Media Strategies in Community-Partnered HIV Research: Practical Considerations From 3 Ongoing Studies

**DOI:** 10.2196/73318

**Published:** 2025-09-10

**Authors:** Elena P Rosenberg-Carlson, Tamra B Loeb, Raquel G Hernandez, Jane J Lee, Aima A Ahonkhai, Jessica M Perkins, Chunqing Lin, Sung-Jae Lee, Sharon Hurt, Luke Johnsen, Alison B Hamilton

**Affiliations:** 1 Department of Family Medicine David Geffen School of Medicine University of California, Los Angeles Los Angeles United States; 2 Department of Psychiatry and Behavioral Sciences Stanford Medicine Stanford United States; 3 Department of Psychiatry and Biobehavioral Sciences David Geffen School of Medicine University of California, Los Angeles Los Angeles United States; 4 Center for Pediatric Health Equity Research Johns Hopkins All Children's Hospital St Petersburg United States; 5 Department of Pediatrics Johns Hopkins Medicine Baltimore United States; 6 School of Social Work University of Washington Seattle United States; 7 Suzanne Dworak-Peck School of Social Work University of Southern California Los Angeles United States; 8 Division of Infectious Diseases Massachusetts General Hospital Boston United States; 9 Center for AIDS Research Harvard University Cambridge United States; 10 TN Center for AIDS Research Vanderbilt University Medical Center Nashville, TN United States; 11 Department of Human and Organizational Development Vanderbilt University Nashville United States; 12 Street Works Nashville, TN United States; 13 Metro Inclusive Health St Petersburg, FL United States; 14 VA Center for the Study of Innovation, Implementation, and Policy VA Greater Los Angeles Healthcare System Los Angeles United States

**Keywords:** HIV, social media, community partner, academic-community partnerships, Ending the HIV Epidemic

## Abstract

**Background:**

In recent years, social media has emerged as a pivotal tool in implementation science efforts to address the HIV epidemic. Engaging community partners is essential to ensure the successful and equitable implementation of social media strategies. There is a notable lack of scholarship addressing the operational considerations for studies using social media strategies in community-partnered HIV research. This article seeks to bridge this gap by consolidating field notes and practical considerations derived from 3 ongoing NIH-supported studies focused on Ending the HIV Epidemic in the United States.

**Objective:**

This article aims to inform the design, planning, and implementation of operationally effective community-partnered social media strategies in HIV research, ultimately contributing to enhancements in HIV practice and improved outcomes across the HIV prevention and care continua.

**Methods:**

Supported by the University of California, Los Angeles Rapid, Rigorous, Relevant (3R) Implementation Science Hub, the 3 Ending the HIV Epidemic projects convened to form the community-partnered social media campaigns working group. The working group used the Consolidated Framework for Implementation Research to help identify and organize key barriers and facilitators of relevance to implementation of the projects’ social media strategies. Given the high degree of interrelatedness across reported factors, the working group thematically synthesized the content into 5 practical considerations to inform use of community-partnered social media strategies in HIV research.

**Results:**

The practical considerations identified by the community-partnered social media campaigns working group include the following: (1) the power and pitfalls of social media platforms (ie, opportunities and challenges inherent to social media platforms that may affect use of social media strategies in HIV research), (2) messengers and messages matter (ie, ensuring the appropriateness, acceptability, and quality of social media messengers and content), (3) the significance of the sociopolitical environment (ie, characterizing the sociopolitical environment surrounding HIV and its potential impact on implementing social media strategies to reach priority populations), (4) investing in academic-community partnerships (ie, cultivating positive and productive academic-community partnerships to support implementation of social media strategies in HIV research), and (5) the alignment of the institutional environment and research approach (ie, assessing and working to address features of institutional environments that may impact implementation of social media strategies in community-partnered HIV research).

**Conclusions:**

As use of social media in HIV research and practice continues to grow, the practical considerations presented in this paper can help research teams anticipate factors that may impact implementation of community-partnered social media strategies and take early action to mitigate potential challenges. By understanding and addressing the unique challenges and opportunities of social media in community-partnered HIV research, we can leverage these platforms to accelerate progress toward ending the HIV epidemic.

## Introduction

Technological advances have transformed the landscape of health interventions, with the use of social media emerging as a pivotal implementation strategy [1]. Social media platforms play an integral role in disseminating information, fostering community engagement and communication, and promoting healthy behaviors [2-4]. In the context of HIV prevention and care, social media is increasingly used to promote awareness and uptake of evidence-based interventions, such as pre-exposure prophylaxis (PrEP) [5,6]. In addition, HIV researchers have leveraged social media in studies aiming to enhance program reach, strengthen coordination across health care sectors, guide clinical decision-making, and improve treatment retention and adherence [7]. Social media is also routinely used as a recruitment tool for HIV-related studies [8,9]. Its capacity to engage diverse populations, particularly those hard to reach due to geographic distance and stigma, underscores the growing relevance of social media in implementation science efforts to address the HIV epidemic [10].

The rapid growth of social media also presents challenges. Platforms such as Facebook (Meta Platforms), Instagram (Meta Platforms), TikTok (ByteDance Platforms), and YouTube (Alphabet Platforms) each have unique characteristics and user demographics, necessitating tailored strategies to connect with target audiences [11,12]. Not all target audiences have equitable access to the internet or familiarity with social media platforms, reflecting a digital divide that can further exacerbate health disparities [13]. Furthermore, protecting the privacy and confidentiality of individuals on social media, particularly in the context of HIV, is paramount. Doing so requires special considerations different from and beyond those typically required in in-person settings [14,15]. The constant evolution of social media technology, algorithms, and policies can also affect the visibility and reach of health intervention content [16]. Involving community partners is essential to effectively mitigate these challenges and ensure successful and equitable implementation of social media strategies in HIV implementation science efforts.

Despite the widespread adoption and implementation of social media, there is a notable lack of scholarship addressing the operational considerations for studies using social media strategies. This article aims to bridge this gap by consolidating field notes from 3 ongoing Ending the HIV Epidemic (EHE) Center for AIDS Research and AIDS Research Center supplement projects. Funded by the National Institutes of Health, these grants focus on enhancing the implementation science knowledge base needed to support the federal EHE initiative using partnered approaches among academic researchers, local health officials, and community groups to address HIV prevention and treatment needs in areas with high HIV transmission [17]. The 3 projects highlighted here each incorporate community-partnered social media strategies in their research to address this public health challenge.

Using the Consolidated Framework for Implementation Research (CFIR) [18] as a conceptual guide, we systematically identified the social media strategy characteristics, outer setting factors, inner setting factors, and processes involved in leveraging social media in each of these ongoing HIV implementation science projects. We aim to assist others engaged in HIV research by identifying common implementation determinants relevant to the use of community-partnered social media strategies across the 3 studies and synthesizing the content into practical considerations that can help researchers anticipate and mitigate potential challenges. Ultimately, these insights are intended to inform the design, planning, and implementation of operationally effective community-partnered social media strategies in HIV research, contributing to enhancements in HIV practice and improved outcomes across the HIV prevention and care continua.

## Methods

### Study Setting

#### Overview

The University of California, Los Angeles Rapid, Rigorous, Relevant (3R) Implementation Science Hub (hereafter referred to as the Hub) is funded by a National Institute of Mental Health EHE supplement award to the University of California, Los Angeles Center for HIV Identification, Prevention, and Treatment Services (grant P30MH058107). The Hub aims to deliver expert technical assistance, coaching, and consultation to funded EHE implementation science projects and develop HIV-related implementation science capacity and collaborations locally, regionally, and nationally. The Hub’s unique goal is to provide leadership and support for rapid, rigorous, and relevant (3R) HIV-related implementation research, emphasizing pragmatic study designs and methods that address health equity and produce sustainable solutions. As part of its routine services, the Hub team facilitates all-awardee meetings to provide opportunities for networking, collaboration, and collective problem-solving among its assigned EHE projects. Through these convenings and with the Hub’s support, 3 projects that were each using community-partnered social media strategies expressed interest in collaborating on a manuscript to share practical considerations and lessons learned. The leads of these 3 projects (RGH and LJ; JJL; and AAA, JMP, and SH) and members of the Hub team (ABH, CL, EPR-C, SJL, and TBL) formed the community-partnered social media campaigns (CPSMC) working group to pursue this collaborative manuscript. Descriptions of each project and their use of social media are provided in the following subsections (summarized in Table 1).

**Table 1:** Summary of community-partnered social media campaign working group projects and their use of social media.

Project	Target population	Project goal	Social media strategy goal	Social media strategy description
A	SGDY^a^ and AYA^b^ in Tampa Bay, Florida	Identify HIV testing and PrEP^c^ deserts and inform the development of equity-focused HIV interventions	Recruit participants for data collection activities	Geotargeted advertisements on Instagram as well as on social dating apps (ie, Grindr and Adam4Adam) and Google Ads
B	Latino and Latinx MSM^d^ in King County, Washington	Implement and evaluate a culturally relevant social media campaign to increase uptake and use of HIV testing and PrEP	Increase uptake of and engagement in HIV services	Instagram and Facebook campaign in partnership with a local Latinx LGBTQ^e^ organization
C	Black men in Shelby County and Davidson County, Tennessee	Promote men’s sexual health and wellness and combat HIV stigma in the Black community	Deliver an intervention to address HIV stigma	Black barbers as men’s health ambassadors sharing curated posts and videos on their social media platforms (X, Facebook, Instagram, and LinkedIn)

^a^SGDY: sexual and gender diverse youth.

^b^AYA: adolescent and young adult.

^c^PrEP: pre-exposure prophylaxis.

^d^MSM: men who have sex with men.

^e^LGBTQ: lesbian, gay, bisexual, transgender, queer.

#### Project A: Using Social Media to Recruit Participants for Data Collection Activities

The Mapping Adolescent and Young Adult HIV Testing and PrEP Deserts within the Tampa Bay Region: Promoting Equity Among Sexual and Gender Minority Youth project (principal investigators: RGH and LJ) assesses existing HIV surveillance data, characterizes adolescent and young adult (AYA)–centered HIV care and prevention services (with a focus on PrEP use), and collects and analyzes perspectives from AYA and sexual and gender diverse youth and other key community stakeholders toward promoting equity in HIV treatment and prevention services. Identification of HIV testing and PrEP deserts (broadly defined as areas where the demand or need for HIV testing among AYAs is greater than the supply of AYA-focused HIV and PrEP sites) [19] is a central study outcome that will inform the development of community and clinical interventions. The project uses geotargeted web-based advertisements to recruit AYA participants for ideation workshops focused on understanding the problem of HIV and PrEP deserts in the Tampa Bay, Florida, area, using three types of media channels:

(1) social media applications (ie, Instagram), (2) social dating applications (ie, Grindr and Adam4Adam), and (3) paid search campaigns (ie, Google Ads).

Specific media channels, including the existing social media channels of the project’s community partner organization, were selected and advertisement content was developed using feedback from community partners to effectively reach the target population. Content included images of individual AYAs, couples, and relevant medical objects (eg, PrEP and HIV tests) as well as messages focused on empowerment and advocacy for sexual health. Social media content required a distinction between static (preapproved, unchanging materials, eg, stock images) and dynamic (interactive or adaptive materials, eg, trend-driven videos) content for institutional review board (IRB) approval. Dynamic content also included the use of contemporary audio or music and pop-culture trends, as well as strategic hashtags to engage local populations.

#### Project B: Using Social Media to Increase Uptake of and Engagement in HIV Services

The Implementation of a Culturally Relevant Social Media Campaign to Increase HIV Testing and PrEP uptake in Latinx MSM project (principal investigator: JJL) harnesses the innovative capacity of social media applications in engaging community members for HIV prevention. The study team is implementing and evaluating a culturally relevant and community-informed social media campaign to enhance uptake of HIV testing and PrEP among Latino and Latinx gay, bisexual, and other men who have sex with men (MSM) in King County, Washington. Project aims include the following: (1) implement the culturally tailored social media (ie, Instagram and Facebook) campaign to facilitate HIV testing and PrEP uptake among Latino and Latinx MSM in partnership with a community organization dedicated to providing HIV and other services to the local Latino and Latinx lesbian, gay, bisexual, transgender, and queer community; (2) evaluate the effectiveness of the campaign in shaping behavioral determinants and HIV testing and PrEP uptake among clients at the organization; and (3) identify the costs and conditions that influence reach, maintenance, and scalability of the campaign to increase HIV testing and PrEP uptake in the Latino and Latinx MSM community.

A community-informed social media campaign was developed specifically for Latino and Latinx MSM and piloted in collaboration with the study’s community partner organization, Entre Hermanos, in a previous study [20]. Guided by the unified theory of behavior [21,22], which identifies determinants of health behaviors (ie, normative pressures, beliefs and expectancies, self-concept and image, affect and emotions, and self-efficacy) [23], the study team developed culturally tailored social media content that addresses salient unified theory of behavior constructs and barriers to HIV prevention identified among Latino and Latinx MSM through qualitative interviews and focus groups [24]. The content reflected their specific needs and preferences, and a pilot study demonstrated the feasibility of the social media content in reaching high-risk Latino and Latinx MSM in King County [20]. For this study, the project team implemented the social media campaign on Facebook and Instagram over a 2-month period in Washington State to evaluate its impact on behavioral predictors of HIV testing and PrEP and HIV testing and PrEP use outcomes among Latino and Latinx MSM who seek services at Entre Hermanos.

#### Project C: Using Social Media to Deliver an Intervention to Address HIV Stigma

The Cutting Out Stigma: A Barbershop-Based Strategy to Reduce Stigma towards Black Men Living with HIV in TN project (principal investigators: AAA, JMP, and SH) involves implementing a barbershop-based health education and multimedia intervention focused on promoting men’s sexual health and wellness and combating HIV stigma in the Black community in the 2 counties in Tennessee with the highest HIV prevalence and incidence—Shelby County (home to Memphis) and Davidson County (home to Nashville). This multipart intervention includes the following: (1) training Black barbers as men’s health ambassadors to foster important conversations about HIV in the context of sexual health and wellness, and to share relevant community resources with barbershop patrons and (2) launching a multimedia HIV stigma reduction campaign involving print, audiovisual, and social media in and around Black barbershops in Tennessee.

The social media strategy comprises 26 messages that first introduced and branded the participating barbers as men’s health ambassadors; provided information about HIV (“Fact Fridays”); provided motivational or educational information about general health in addition to sexual and mental health and wellness; and provided opportunities for barbers to create and share their own live videos (“Cuts and Convos”), pictures, and text (“Choose Your Style, Choose Your Health”). Participating barbers were asked to post curated messages at least once weekly on their social media platforms (X, Facebook, Instagram, and LinkedIn). Barbers were advised to post on prespecified days and times and to use tailored hashtags to optimize campaign reach. Where possible, barbers also were asked to collaborate with or tag the study team’s social media account to further promote the reach of the messaging. The last component of the social media strategy involved the creation of short videos that feature some of the participating barbers in a local barbershop discussing men’s sexual health and HIV. Barbers play these short videos on the screens in their barbershops at least 3 times weekly and share them via social media. The goal of the social media strategy and broader multimedia campaign is to spark conversations and engagement regarding HIV and HIV-related stigma, and to help reframe the stigmatizing narrative about HIV in the community.

### Data Collection

Each project used constructs from the CFIR [18] in its initial project logic model to describe contextual factors germane to its planned project strategies and activities. Given the comprehensive nature of the CFIR and its preexisting application across projects, we used the CFIR innovation, outer setting, and inner setting domains as conceptual anchors and the domain constructs as a framework to help identify and organize key barriers and facilitators of relevance to implementation of the projects’ social media strategies.

The academic project leads of the 3 projects (RGH, JJL, and AAA and JMP) began by identifying constructs relevant to their respective projects within the CFIR innovation, outer setting, and inner setting domains and describing how these constructs were operationalized and addressed in the context of implementing their social media strategies. During this process, academic project leads consulted with other members of their research teams as needed to ensure complete identification of key constructs. Academic project leads then engaged in informal conversations with their respective community partners to elicit and document their experiences with strategy implementation in the context of a research process involving academic-community partnerships. Community partners discussed barriers to and facilitators of strategy implementation, as well as benefits and challenges of participating as community partners in the projects. All original insights represented in this paper reflect this within-project examination of the experiences and perspectives of project team members, including academic and community partners engaged in research and implementation activities.

### Data Analysis

The initial constructs identified by academic project leads were compiled in a Microsoft Excel matrix and reviewed for common constructs across the projects by the Hub project director (EPR-C). The matrix and common constructs were presented to the CPSMC working group and reviewed, discussed, and refined over 2 CPSMC working group meetings, as well as through opportunities for individual review and written feedback. Given the high degree of interrelatedness across CFIR constructs and reported factors, after a set of common constructs was compiled, 2 Hub team members (EPR-C and TBL) worked together to thematically synthesize the content into preliminary practical considerations for use of community-partnered social media strategies in HIV research.

Notes from the individual conversations between academic project leads and community partners were reviewed and assessed by EPR-C and TBL for concordance with the preliminary practical considerations. The practical considerations were then modified to integrate community partner perspectives and experiences. The modified set of practical considerations was then reviewed over 2 CPSMC working group meetings, opportunities for individual review and written feedback, and iteration by EPR-C and TBL until consensus was reached.

### Ethical Considerations

The 3 projects discussed in this paper are considered research and were, respectively, reviewed and approved by the Johns Hopkins University School of Medicine IRB (approval #IRB00377619; project A), the University of Washington IRB (approval #STUDY00015837; project B), and the Vanderbilt IRB (approval #240024; project C). Ethics approval and consent to participate is not applicable to the initiative described in this paper.

## Findings

### Overview

The five practical considerations identified by the CPSMC working group to inform use of community-partnered social media strategies in HIV research include the following: (1) the power and pitfalls of social media platforms, (2) messengers and messages matter, (3) the significance of the sociopolitical environment, (4) investing in academic-community partnerships, and (5) the alignment of the institutional environment and research approach. Each consideration and relevant subconsiderations are detailed in the following subsections in the form of synthesized reflections and recommendations from the project teams, drawing on relevant examples from the 3 projects’ experiences in the field.

### Consideration 1: The Power and Pitfalls of Social Media Platforms

Numerous opportunities and challenges inherent to social media platforms may affect the use of social media strategies in HIV research. While each social media platform has unique characteristics that may impact its appropriateness for a given project, our focus is on highlighting considerations relevant to the use of any platform.

#### Platforms and User Preferences Constantly Evolve

Each social media platform has unique features that evolve over time, continuously interacting with evolving user preferences and business environments. Shifting cultural trends, social dynamics, and platform features perpetually influence the preferences of diverse and heterogeneous user groups. Due to these constant shifts, with new trends continuously emerging and others dissipating, social media strategies that resonate with a particular community today may be less impactful tomorrow.

Ongoing engagement with the community is essential to stay attuned to these changes and to ensure that a campaign remains responsive and effective. For example, in project B, Latino and Latinx MSM community members suggested using particular hashtags and emojis of current relevance to best connect with their community. Notably, keeping pace with user preferences and behaviors requires a significant investment of time and resources. For these 3 projects, ensuring the continued relevance of particular social media approaches demanded ongoing monitoring and adaptation, which may be constrained by time, personnel, and funding. Moreover, evaluating the impact of social media strategies amidst this dynamic environment poses further obstacles, as the metrics and benchmarks for success may themselves undergo rapid transformation.

#### Projects May Be Limited by Platform Policies and Guidelines

Social media platforms face their own hurdles in managing the evolving landscape of user-generated content, sometimes resulting in the prohibition of content and social media strategies that particularly resonate with target communities. For example, project B noted that they were blocked from posting the content and images that were most popular with the Latino and Latinx MSM community they were trying to reach, posing a major barrier to implementation of their campaign. Furthermore, platform guidelines are constantly changing and may prohibit specific text and images that were previously acceptable for dissemination, as experienced by project B, potentially necessitating adaptations to planned content. Each social media platform also has unique content specifications, such as word count limits; varying levels of reliance on static images, videos, and text-based content; and restrictions on use of hyperlinks that must be considered in assessing its appropriateness for a given project.

#### Use of Social Media Introduces Unique Data Use and Security Complexities

Researchers must be aware of the potential risks to the privacy and confidentiality of participants inherent in using these platforms, as the evolving nature of social media introduces uncertainties regarding data use, security, and participant anonymity. Limited guidelines exist for how to best address these risks in the context of research, but individual projects should consider potential ways to mitigate and acknowledge these risks as they develop and implement their use of social media. The CPSMC working group projects highlighted the importance of early communication with IRBs regarding recommended practices for addressing data use and security concerns to avoid extended delays in IRB review and project implementation.

#### Reaching Your Population of Interest With Social Media

Social media is a powerful tool that can facilitate opportunities to reach populations that have not been effectively reached through traditional clinical and public health outreach, particularly given the widespread popularity and accessibility of social media platforms in the United States. All 3 CPSMC working group projects emphasized the importance of assessing and addressing the unique needs and preferences of their populations of interest in identifying the appropriate platforms and approaches to most effectively reach them. Furthermore, while social media can facilitate reach to large and diverse communities, a disconnect may exist between the intended audience and actual message recipients. Factors such as algorithmic biases, user engagement patterns, and content sharing dynamics can influence who ultimately receives and interacts with the content. Platforms may offer some targeting options, but they may not always be as granular as desired to reach particular groups (eg, MSM and trans women). Even when these features exist, some individuals might not be open about their identities nor willing to disclose their identities on social media platforms. These limitations may add another layer of complexity to the implementation of social media campaigns for specific communities. While social media platforms may support reaching certain populations, the level of specificity available for targeting may not adequately align with project goals.

The high potential reach of social media platforms may also pose particular challenges for community partners. For example, project C’s men’s health ambassadors initially raised concerns about how far their messaging may go on social media given their local environment of HIV-related stigma and discrimination. Conversely, while project B’s community partners highlighted their excitement about potentially removing the geographic boundaries of their impact via the use of social media, they were not able to do so in practice because their organization only had the capacity to provide local services.

#### Social Media Is Increasingly Part of Routine Operations in Community Organizations

All 3 projects identified increased use of and investment in social media among community partner organizations in recent years. For example, the clinical organization partnering with researchers on project A noted that they have increased staff dedicated to social media as well as enhanced attention to metrics on the impact of their social media channels. Likewise, the organization has increasingly relied upon social media for outreach to populations of interest, including the AYA populations of focus in project A. The prevalent use of social media by community organizations may help facilitate the use of social media strategies in community-partnered HIV research.

### Consideration 2: Messengers and Messages Matter

#### Identifying Your Messengers

The messengers a research team uses to deliver their social media strategy can have a pronounced impact on strategy success. All 3 CPSMC working group projects gave significant consideration to identifying messengers who would draw the attention and trust of the communities they were seeking to reach. Messengers may include individuals represented as messengers in social media images as well as the message poster or source. For example, project A clearly indicated the source of the content and visibly displayed the logo or name of the trusted partner organization in social media images to help support engagement. The CPSMC working group projects highlighted that if social media messages appear to be coming from a source that is not accepted, relevant, and credible in the eyes of the target audience, the audience is unlikely to positively engage with the content.

#### Involving Social Media Experts to Ensure High-Quality Content

Social media expertise is a crucial element of successful social media strategy implementation. It may be provided by members of the project team or by external contracted entities. CPSMC working group members noted that public communications and social media education are typically not included in the traditional investigative training pathway, which may result in projects needing to rely more heavily on external expertise. When possible, it is useful to work with experts in social media who have experience communicating about HIV or other potentially sensitive health topics to the population of interest. For example, project C contracted with a Black-owned media and marketing company with expertise in health interventions to help develop their HIV stigma reduction multimedia campaign. As one of their men’s health ambassadors noted, “Social media marketing can be effective if it’s done really well or people will remove it, block it.”

#### The Importance of Community Input

Community partners are necessary opinion leaders, champions, and external change agents. All 3 projects emphasized that any HIV-focused study intending to implement social media strategies should consider how best to include community partners who reflect and are meaningfully connected to the population they are working to reach throughout their strategy development and implementation process. Community input on social media strategies may also be gathered via focus groups with members of the population a study intends to reach (as was done in project B), pilot-testing, and other idea generation and feedback opportunities with community members. Community partners involved in project B emphasized that “overall, community members were responsive to our campaign because it was designed for Latinos by Latinos.” The 3 projects stressed that the quality of social media messaging reflects the care that was taken to design the message to reach, connect with, and inform the population of interest, which requires community input.

While community input is crucial and may offer consistent guidance on certain elements of a social media strategy, it is also important to acknowledge the heterogeneity in communities. All 3 projects observed diverse community member opinions that did not always lead to consensus about the preferred look and feel of their social media content. For example, project A shared that photographs of local youth self-reporting as sexually or gender diverse within an Instagram campaign were considered by some community partners to be denoting too much vulnerability and not demonstrating empowerment. Likewise, project B noted that some participants desired images that “looked like them,” which was a key element of their content development process, but given the diversity within the Latino and Latinx MSM community, not everyone always felt represented. Still, all 3 CPSMC working group projects agreed that a community-engaged development process was invaluable to refining messages and identifying trusted messengers.

#### Your Content in Context

The context in which an HIV social media strategy is implemented has major implications for how content may be received. The high potential reach unique to the use of social media strategies can pose challenges in contexts where HIV is highly stigmatized. As a men’s health ambassador involved in project C shared, some people in their local area of Tennessee “don’t want to be attached to [HIV] and don’t want to be bothered.” While the potential to reach a large audience is generally advantageous for spreading awareness and disseminating information, it may pose additional challenges for research efforts navigating sensitive topics in environments marked by stigma and discrimination. To minimize this risk, the CPSMC working group projects emphasized that researchers using social media strategies should use inclusive and empowering content that presents communities in ways that align with how they desire to be portrayed, being careful to avoid perpetuating stigma.

Relatedly, an important piece of guidance that arose from community input in these projects was to avoid negative framing of disparate health outcomes. Community members emphasized that images and text should have a positive tone that avoids stereotypes or foreboding messages. Paying attention to communities’ strengths and resilience may be particularly important for social media strategies focused on marginalized groups, especially in contexts where there is a high level of stigma related to HIV and sexual health. Research teams should also anticipate potential mistrust by community members around targeting specific populations. Studies may need to actively work to establish trust, demonstrate trustworthiness, and consider historical harms in communities of interest to come to a place where their content can be positively received and considered credible by community members.

### Consideration 3: The Significance of the Sociopolitical Environment

The sociopolitical environment surrounding HIV and its effect on implementing social media strategies to reach priority populations can have a tremendous impact on project success. To illustrate the importance of considering the sociopolitical environment, these dynamics are explored in the context of each project in the following subsections.

#### Project A: Social Media as a Recruitment Strategy When Traditional Strategies Are Nonviable Due to Local Attitudes Toward the Target Population

Lesbian, gay, bisexual, transgender, and queer individuals in Florida, as in other states, face worsening disparities in health care access and treatment that heavily influence the implementation of HIV-focused research. Sexually and gender diverse youth within the state are particularly susceptible to severe discrimination, lack of culturally competent care, and marginalization as past HIV-focused treatment and prevention programs were largely closed or minimized in the wake of legislative measures, including the “Don’t Say Gay” bill [25] as well as a ban on gender-affirming treatment for youth [26] passed within the state in 2023. As a consequence of these sweeping policy changes, traditional recruitment strategies, such as in-person discussions, use of flyers, and brick-and-mortar-based information, were no longer available for project A, which focused on the inclusion of sexually and gender diverse youth. Furthermore, within the pediatric health care organization involved in the project, clinical programs, such as the adolescent medicine and gender-affirming care clinic, were closed, and clinical and research teams were subject to personal threats for providing care to this population. With local and regional channels to reach and support this highly vulnerable population suspended, social media–based recruitment became the primary and safest method for outreach for project A. This recruitment strategy, while limited to the structure and scope of public-facing platforms, offered the only way by which to communicate information related to sexual health to populations at risk for HIV in a way that protected content from new laws and processes that limit traditional “free speech” opportunities available in other states to engage diverse populations in HIV-focused research.

#### Project B: Community Acceptability and Supportive Policies Engender a Welcoming Atmosphere for a Social Media Campaign

Project B’s decision to develop a social media campaign for Latino and Latinx MSM via their academic-community partnership stemmed from the community’s receptiveness to technology-based outreach strategies for HIV prevention. The popularity of social media, coupled with expanded efforts to increase HIV prevention in Washington State and particularly in King County, created a welcoming atmosphere for public initiatives to encourage use of HIV-related services. Specifically, Washington has supportive policies that help eligible people with HIV obtain medications as well as PrEP drug assistance programs that provide financial assistance for people interested in accessing PrEP. Project B’s community partner organization was also involved with learning collaboratives as part of King County’s EHE efforts, which support health care organizations across the county in better identifying and serving people at risk for HIV. These initiatives demonstrated a readiness and favorable environment for project B and helped facilitate the implementation of its social media campaign.

While perceptions of HIV vary widely across different communities and counties within Washington, progressive policies and supportive programs may contribute to a relatively accepting environment for individuals living with or at risk for HIV. Thus, social media strategies and interventions for HIV prevention may be more feasible in Washington compared to places with more restrictive environments.

#### Project C: A Social Media Strategy to Address HIV Stigma Plans for Stigma-Based Community Reactions

The broad sociopolitical environment in Tennessee is characterized by various legal and cultural factors that act as barriers to addressing HIV. While pervasive HIV-related stigma has been identified as a key contributor to the disproportionate effects of HIV across the Deep South [27], in Tennessee, this stigma has manifested in some of the most stringent HIV-related criminalization laws in the country. For example, until July 2024, Tennessee was the only US state that imposed a lifetime registration as a “violent sex offender” if convicted of engaging in sex work while living with HIV [28].

Over the last several years, Tennessee has made gradual progress on key HIV prevention and treatment outcomes. However, recent political decisions indicative of continued stigma around HIV and populations most impacted by HIV have the potential to reverse this progress. In 2023, Tennessee rejected millions of federal dollars for HIV prevention, while simultaneously funding prevention efforts from the state’s budget and prioritizing new populations (pregnant people, survivors of sex trafficking, and first responders) over populations with the greatest risk and burden of HIV in the state [29]. In the context of project C, the stigma and fear culturally associated with HIV were carefully considered during each step of the social media strategy design and implementation process. In addition, the barbers who became men’s health ambassadors as part of the project expressed that social media amplification of the campaign would make some individuals in their communities assume that the barbers have HIV. They said that preparation for how to challenge this assumption would be useful and appreciated. In this way, the broad potential reach of social media was a source of potential concern for project C’s strategy implementers who were aware they may become targets of stigma and discriminatory behavior if assumed to have HIV.

### Consideration 4: Investing in Academic-Community Partnerships

#### Identifying the Right Partners

In any academic-community research partnership, cultivating a positive relationship, establishing shared project goals, and identifying the contributions from and benefits for both partners is paramount. For researchers aiming to implement HIV-related social media strategies, this process starts with identifying a community organization that is experienced with and sensitive to HIV-related topics, and trusted by and, where possible, culturally congruent with the population to be reached. It is also helpful for the organization to have social media and communications expertise, ideally specific to reaching the priority population with health messaging. Having a strong previous working relationship with the organization and its leadership is very useful, as was the case with projects A and B, or the study team must invest in building that strong working relationship in preparation for strategy implementation, as in project C [30].

#### Building Research Literacy

Many community organizations conduct effective programmatic work with populations highly impacted by HIV and have limited knowledge of research. Among the community partners working on these 3 projects, there was variable familiarity and experience with research. For example, while project A’s community partner organization, a clinical setting, had previously participated in research, a member of their clinical leadership team shared that “research is just not built into the organization.” He also described receiving “blank stares when we talk about data.” The CPSMC working group projects noted that researchers seeking to start or strengthen partnerships with community organizations should be prepared for a potential lack of familiarity, comfort, and interest in research among staff. Likewise, key elements of research projects, such as administration of grant funding, project roles, and project timelines, may not be intuitive to community partner staff, which may cause challenges during project initiation and implementation. Sharing the research purpose and components and outlining the practical, administrative elements of project implementation with community partners early on will help build research literacy and lay the foundation for a successful academic-community partnership.

#### Communicating Effectively

With all partnerships, and particularly when working with community organizations newer to research, it is key for the academic partner to provide clear, transparent, and tailored communication that meets the community partner organization where they are. The academic team should endeavor to communicate the relevance and intended impact of the project as it relates to the community organization’s work and avoid using technical jargon. In addition, routinely inviting and responding to questions from community partners is important. All 3 CPSMC working group projects noted that trust is at the core of any positive partnership, and therefore, communication between partners should be timely and reliable.

#### Pursuing the Win-Win

Often, academic-community partnerships are developed primarily to serve research-related goals set forth by the academic partner. Funds are typically provided to community partners via subcontract to support their participation. However, projects A and C noted that in practice, it can remain challenging to add research activities to a staff person’s plate outside of their routine role. A member of the clinical leadership team for project A’s community partner organization also noted it can be tricky to assign and supervise research-related roles among existing staff, asking “How do I give feedback on a role they weren’t hired for?”

Given these challenges, it is crucial for researchers to work to identify and support benefits of participation for community partners. Researchers may consider eliciting information from staff at their partner organization about what they hope to gain from participating. For example, becoming men’s health ambassadors was seen as a growth opportunity for some of the barbers participating in project C. One commented they wanted to be “a part of something greater” by participating in this project to improve HIV-related outcomes. Some also expressed concern about not being integrated into future efforts after “building a team of believers” through this work, presenting an important opportunity for the academic team to consider how to keep them engaged as long-term champions. Ensuring allocation of appropriate financial incentives for community partners is also key, particularly for those who may not have consistent wages paid by the organization, as was the case in project C.

### Consideration 5: The Alignment of the Institutional Environment and Research Approach

#### Overview

The 3 CPSMC working group projects unanimously agreed that while academic-community partnerships have increasingly been used in HIV research to facilitate the development of interventions and strategies tailored to the unique needs of communities most impacted by HIV, this approach remains unfamiliar to many academic institutions and their IRBs. All 3 projects experienced tension between the increased national priority for HIV researchers to conduct community-partnered research and institutional structures that were not created with community-partnered research frameworks in mind nor designed to support community-partnered research. The projects reported struggling with IRB-related policies and infrastructures that hinder the implementation of timely, bidirectional community-partnered research. Additional challenges included institutional restrictions and limited guidance regarding implementing social media strategies as part of these research endeavors. This section highlights the importance of assessing and working to address features of institutional environments that may impact implementation of social media strategies in community-partnered HIV research, exemplified by the experiences of the CPSMC working group projects.

#### Assessing Institutional Preparedness to Support Your Research

All 3 projects described experiencing significant institutional constraints and generally conservative approaches by institutional IRBs when reviewing their community-partnered HIV research protocols. They commented that despite community engagement and partnership increasingly being included as required elements of proposals for HIV research funding nationally, community-partnered research methodologies are still considered novel and are misaligned with the research infrastructures of many academic institutions. For example, they reported challenges with the inclusion of community partners as “coinvestigators” on IRB protocols, limiting true partnership and coownership of studies. They also highlighted a lack of resources and support from IRBs to help community partners become familiar with institutional processes and requirements, such as training modules on human research protections tailored for community partners.

These challenges were heightened when combining a community-partnered HIV research approach with social media strategies. Although most IRBs are well-attuned to supporting traditional recruitment strategies, projects noted that there appear to be few precedents as to how social media and other digital platforms could be used to connect with potential participants while still protecting participant privacy and confidentiality. In addition, because user interactions with content feed each social media platform’s private algorithms that shape future content displays, participants may receive future information on the platform that is uncontrolled by and unpredictable for the research team and their institutional IRB. This uncertainty may create potential challenges for the IRB’s efforts to ensure human participants’ protection and sometimes results in significant constraints on the research team’s social media strategy design and implementation. Furthermore, the CPSMC working group projects found their IRBs to be generally unfamiliar with the use of nonstatic content (eg, videos and reels). They noted that IRBs often do not have guidelines or systems for assessing these dynamic forms of information sharing, which are increasingly popular among social media users. Although social media–based approaches to recruitment, engagement, and intervention delivery are increasingly used in research and have been highly effective in reaching populations most impacted by HIV, the experiences of these 3 projects suggest that institutional IRBs may be underprepared to support implementation of social media strategies.

#### Navigating the Impact of Your Institutional Environment on Your Research

These challenges place community-partnered HIV research projects implementing social media strategies in situations where projected timelines are almost certain to be delayed pending their institution’s readiness to provide relevant support and guidance for their research approach. Two of the 3 CPSMC working group projects experienced major IRB-related delays that halted important study progress for several months. Crucially, these institutional challenges and resulting delays can create delays in payment and confusion and frustration among community partners.

The projects noted that some of this frustration among community partners may have stemmed from a lack of understanding about the function of IRBs and how they work, further reflecting the need for academic partners to invest in building research literacy among all members of their project teams. Likewise, some partners were not familiar with the risks and responsibilities of engaging in human participants research and complying with IRB requirements. Certain requirements were also found off-putting by community partners, such as the requirement to engage in an informed consent process using a document that contained standard IRB language that felt “uncomfortable” or “unnatural.” Compounded with restrictions and delays that hindered project progress, project C noted that this discomfort resulted in some community partners questioning the intentions of and need to rely on academic institutions. One partner from project C asked, “Do we always have to wait on something to make a difference?” and “Do we always need some outside source for the community, or do we have what we need?”

The CPSMC working group projects came to consensus in emphasizing that academic partners are ultimately responsible for ensuring community partners are equipped to adhere to institutional policies and procedures and are prepared for administrative processes that may cause delays in project activities and, in some cases, payment to community partners. Given the observed lack of resources and support from IRBs to help community partners become familiar with institutional processes and requirements, researchers must be prepared to provide education about what to expect with respect to the academic institution; subcontracting processes; and the purpose, policies, and procedures of the IRB that are relevant to community partner roles in the project. Researchers should also be prepared to proactively advocate for the needs of their community partners in an attempt to support strong and sustainable academic-community partnerships.

## Discussion

### Implications of Findings

The federal EHE initiative has brought renewed attention and investment to addressing the HIV epidemic in the United States. A “whole-of-society” initiative, EHE emphasizes innovative, community-engaged approaches to optimizing HIV research and practice [31]. In community-partnered HIV research, social media is increasingly used as a strategy to reach potential participants and deliver interventions [5-7]. However, it remains a newer approach, and there is little practical guidance available for HIV researchers interested in using social media strategies. This article features the voices and experiences of 3 research teams and their community partners, offering real-world, pragmatic insights from their projects that are more likely to be shared informally among the research community than published in peer-reviewed journals. These insights provide essential guidance to inform the effective implementation of community-partnered social media strategies in HIV research. Five practical considerations were identified, addressing the unique nexus of HIV research, academic-community partnerships, and social media strategies.

While we intend for these notes from the field to offer pragmatic guidance for researchers, this article also serves to spotlight salient implementation barriers commonly identified across 3 distinct HIV research projects. Despite previous calls to address the discordance between institutional structures and community-partnered research approaches [32], the experiences of these 3 projects demonstrate that there remains an urgent need to develop solutions to this ongoing challenge. The barriers experienced by the CPSMC working group projects are largely consistent with those identified in other recent literature. For example, a scoping review of 15 US-based community-engaged research projects by Onakomaiya et al [33] described four key challenges related to working with IRBs: “(1) community partners not being recognized as research partners; (2) cultural competence, language of consent forms, and literacy level of partners; (3) IRBs apply formulaic approaches to community-engaged research; and (4) extensive delays in IRB preparation and approval potentially stifle the relationships with community partners.” While there is increased emphasis on community-engaged methods by academic institutions and federal funders, as evidenced by their calls for community-partnered research applications, this ongoing misalignment reflects a culture and infrastructure that are not fully prepared to support community-partnered research. Furthermore, as identified by these 3 projects, these barriers limit researchers’ abilities to build strong, sustainable relationships with community partners. Educating IRBs on community-partnered research methodologies, revisiting policies that create barriers to community-partnered research [34], and developing and disseminating human participants research training modules tailored for community partners [35] could help bridge the gap between current institutional infrastructures and community-partnered research approaches.

Likewise, IRBs have limited preparedness to assess and support projects using social media strategies. As noted by the CPSMC working group projects, use of social media in human participants research has raised new ethical and regulatory complexities, and there remains limited guidance for IRBs from federal regulatory bodies. In the absence of that guidance, IRBs and researchers have a responsibility to carefully consider the unique participant privacy and autonomy implications of using social media strategies, balanced with the immense positive potential of using these tools in research endeavors. While protecting research participants is important for engaging in responsible research activities, we are potentially losing creative, innovative, and effective approaches to help end the HIV epidemic by not optimizing the implementation of social media strategies in HIV research. The rubric proposed by Bhatia-Lin et al [36] to assist studies to determine ethical and regulation-consistent use of social media platforms may offer a useful framework for these considerations.

More broadly, the insights shared in this paper highlight the need to continue working toward addressing the historical separation between academia and the community and creating a culture that values and respects building and maintaining strong community partnerships. In the context of implementing social media strategies in community-partnered HIV research, partnership with the community is particularly essential for engaging trusted messengers and developing culturally relevant messages. Likewise, it is critical for research teams to proactively address potential communication and research literacy barriers that may hinder the full realization of their community-partnered HIV research and its potential impacts.

While this article provides practical considerations and guidance regarding implementation of social media strategies in community-partnered HIV research, future research is needed to add to the evidence base on best practices for using social media in HIV research and to continue expanding the growing body of literature on community engagement and partnership in HIV research [37]. Further study is also needed to characterize the unique features of various social media platforms and types of content for use in HIV research, and to assess the effectiveness of community-partnered social media strategies in improving HIV-related outcomes. In addition, developing approaches projects can use to design their social media strategy to match their particular context and research aims would be a useful contribution to the HIV research field and beyond. Though this article focuses on HIV-related research, we also anticipate many of the insights shared here would be transferable to investigators implementing social media strategies and conducting community-partnered research in other fields.

This article offers practical considerations in the form of notes from the field derived from 3 ongoing studies. While instructive, these considerations do not comprehensively represent the many types of social media platforms, content, and strategies used in community-partnered HIV research and are limited to the experiences of the 3 CPSMC working group projects. In addition, we do not capture the effectiveness of these strategies as part of this paper, as all investigations are ongoing. Exemplars of effectiveness studies are available in the literature [38,39] and can provide useful guidance for determining appropriate outcomes for social media strategies.

### Conclusions

As use of social media in HIV research and practice continues to grow, the practical considerations presented in this paper can help studies anticipate factors that may impact implementation of community-partnered social media strategies and take early action to mitigate potential challenges. The potential of social media to reach and engage populations most impacted by HIV is significant, and ongoing research is needed to harness its potential. By understanding and addressing the unique challenges and opportunities of social media in community-partnered HIV research, we can leverage these platforms to accelerate progress toward ending the HIV epidemic.

## Abbreviations

3R: University of California, Los Angeles Rapid, Rigorous, Relevant
AYA: adolescent and young adult
CFIR: Consolidated Framework for Implementation Research
CPSMC: community-partnered social media campaigns
EHE: Ending the HIV Epidemic
IRB: institutional review board
MSM: men who have sex with men
PrEP: pre-exposure prophylaxis
